# Comparative Transcriptome Profiling Reveals the Genes Involved in Storage Root Expansion in Sweetpotato (*Ipomoea batatas* (L.) Lam.)

**DOI:** 10.3390/genes13071156

**Published:** 2022-06-27

**Authors:** Weihan Song, Hui Yan, Meng Ma, Meng Kou, Chen Li, Wei Tang, Yicheng Yu, Qixian Hao, Thanhliem Nguyen, Xin Wang, Zhenyi Zhang, Chang You, Runfei Gao, Yungang Zhang, Qiang Li

**Affiliations:** 1 Xuzhou Institute of Agricultural Sciences in Jiangsu Xuhuai District, Key Laboratory of Biology and Genetic Breeding of Sweetpotato, Ministry of Agriculture and Rural Affairs, Xuzhou 221131, China; xzsongweihan@126.com (W.S.); yanhui_sweetpotato@163.com (H.Y.); brave_ma_2022@163.com (M.M.); koumeng2113@163.com (M.K.); 1020180010@jsnu.edu.cn (C.L.); tangweilhr@163.com (W.T.); xznkywx@163.com (X.W.); zhangzy990813@126.com (Z.Z.); yclucky2022@126.com (C.Y.); 15852141027@163.com (R.G.); 2 School of Life Science, Jiangsu Normal University, Xuzhou 221116, China; z888822984@163.com; 3 State Key Laboratory of Crop Genetics and Germplasm Enhancement, Research Center of Jiangsu Plant Gene Engineering, Nanjing Agricultural University, Nanjing 210095, China; 2020201049@njau.edu.cn; 4 Department of Biology and Agricultural Engineering, Quynhon University, Quynhon 590000, Binhdinh, Vietnam; nguyenthanhliem@qnu.edu.vn

**Keywords:** sweetpotato, storage root expansion, transcriptome, gene, transcription factor

## Abstract

Sweetpotato (*Ipomoea batatas* (L.) Lam.) is recognized as one of the most important root crops in the world by the Food and Agriculture Organization of the United Nations. The yield of sweetpotato is closely correlated with the rate of storage root (SR) formation and expansion. At present, most of the studies on sweetpotato SR expansion are focused on the physiological mechanism. To explore the SR expansion mechanism of sweetpotato, we performed transcriptome sequencing of SR harvested at 60, 90, 120, and 150 days after planting (DAP) to analyze two sweetpotato lines, Xuzishu 8 and its crossing progenies named Xu 18-192, which were selected from an F_1_ segregation population of Xuzishu 8 and Meiguohong, in which SR expansion was delayed significantly. A total of 57,043 genes were produced using transcriptome sequencing, of which 1312 were differentially expressed genes (DEGs) in four SR growth periods of the sweetpotato lines. The combination of the KEGG and trend analysis revealed several key candidate genes involved in SR expansion. The *SBEI* gene involved in starch metabolism, and transcription factors *ARF6*, *NF-YB3* and *NF-YB10* were all significantly up-regulated during SR expansion. The data from this study provide insights into the complex mechanisms of SR formation and expansion in sweetpotato and identify new candidate genes for increasing the yield of sweetpotato.

## 1. Introduction

Sweetpotato (*I. batatas* (L.) Lam.) is both a grain crop and economic crop worldwide, and plays an important role in food security and economic development in China. Sweetpotato SR is the main organ of harvest, its development and expansion directly affect the yield of sweetpotato [1]. The phenotype and cytological structure of SR formation and expansion in sweetpotato were studied in detail. The vine of sweetpotato serves as a propagating material which will produce adventitious roots at the cutting point or stem node after 2–3 days of cutting propagation [2]. At present, sweetpotato root development is generally divided into two stages: the first stage is fibrous root, also known as adventitious root. At first, the root primordia of the sweetpotato stem node gradually differentiate and develop into adventitious roots. The second stage is root tuber, also known as SR. The fibrous roots continuously thicken and produce secondary growth, finally swelling to form SR [3]. While very important, only a few studies have been conducted on its molecular mechanism.

The formation and expansion of SRs in sweetpotato are affected by many environmental and internal factors. The environmental factors mainly include temperature, light, moisture, fertilization levels, and plastic film [4,5]. The internal factors include metabolites, endogenous hormones and related genes. The main metabolites are starch, soluble sugar, protein, and essential amino acids. The endogenous hormones mainly implicated are cytokinin (CTK), abscisic acid (ABA), auxin indole-3-acetic acid (IAA), gibberellin (GA) and jasmonic acid (JA) [6,7].

With the development of modern biological techniques and sequencing of the reference genome of sweetpotato wild species *I. trifida* and the hexaploid cultivar, SR expansion-related genes were identified and cloned [8]. The expression patterns of MADS-box genes *IbMADS79*, *IbAGL17* and *IbAGL20* were significantly different during SR expansion, suggesting that these three genes may be involved in the SR expansion of sweetpotato [9]. *Storage root formation 1* (*SRF1*) is a Dof-like zinc finger protein transcription factor. The overexpression of *SRF1* significantly increased dry matter content and starch content in sweetpotato SR, and positively regulated SR expansion [10]. *SRD1* (*storage root development-related 1*) is a MADS-box transcription factor which is 99% homologous with *IbMADS1*, which is involved in IAA metabolism, and activated the proliferation of cambium cells to regulate the thickening of SRs [11]. The expansion gene *IbEXP1* plays a negative role in SR expansion by inhibiting the proliferation of xylem and cambium cells [12]. A NAC transcription factor, *IbNAC083,* was identified using dynamic network biomarker (DNB) analysis; it acts as a core regulator of SR expansion initiation in the DNB- related network [13]. The overexpression of AP2/ERF transcription factor *IbRAP2.4* significantly inhibited SR formation of transgenic sweetpotato, suggesting that *IbRAP2.4* may be one of the potential targets for high-yield molecular breeding of sweetpotato [14].

Transcriptome sequencing can directly analyze the genes of most organisms in one specific functional state, and does not need to know the genetic information of the plant species. Currently, in sweetpotato, transcriptome sequencing has become a powerful tool to excavate the genes involved in various molecular regulatory mechanisms [15,16]. To date, however, there has only been a few transcriptome studies on SR expansion in sweetpotato. Recently, two labs reported SR expansion research using transcriptome analysis. In their study, RNA sequencing (RNA-Seq) analysis concentrated on different SR expansion stages of one sweetpotato variety or the comparative transcriptomes analysis of the root between cultivated sweetpotato and wild species were investigated [13,17]. Unlike previous studies, in this project we created crossing progenies material through hybridization and used transcriptome sequencing to search for functional genes that affect SR expansion in sweetpotato. Therefore, it has significant innovation and application value in the field of sweetpotato.

In this study, we measured the weight of SRs and performed RNA-seq analysis of two sweetpotato lines: Xuzishu 8 (XZ8) and Xu 18-192 (crossing progenies, X192), at four SR expansion stages (60, 90, 120, and 150 DAP, named SR1, SR2, SR3 and SR4, respectively). These analyses aimed to identify key genes and pathways involved in SR expansion and investigate the molecular mechanism underlying SR expansion in sweetpotato. The findings provided a theoretical basis for the mechanism of SR expansion and breeding of high quantity varieties in sweetpotato.

## 2. Materials and Methods

### 2.1. Plant Materials and Growth Conditions

Sweetpotato cultivar Xuzishu 8 (*I. batatas* (L.) Lam, XZ8) was a purple-fleshed sweetpotato variety newly cultivated by our team. The SR expansion of sweetpotato material Xu 18-192 (crossing progenies, X192) was delayed significantly, when selected from an F_1_ segregation population of XZ8 and Meiguohong. The two sweetpotato lines were planted in the modern agricultural experimental farm of the Xuzhou sweetpotato research center on 26 May 2021. The experiment materials were randomly arranged with 5-row plots, of which there were 10 seedings in each row and three replicates. The plot area was 20 m^2^ with row spacing of 0.85 m and a row length of 4.50 m. The SR of XZ8 and X192 was harvested at 60, 90, 120, and 150 DAP, then washed and photographed before being cut into small pieces. All the samples were rapidly frozen in liquid nitrogen and stored at −80 °C for RNA extraction.

### 2.2. RNA Extraction, cDNA Library Construction and Transcriptome Analysis

Total RNA from the SR samples was extracted using a Trizol reagent kit (Invitrogen, Carlsbad, CA, USA) following the manufacturer’s protocol. The RNA purity and concentration were assessed on an Agilent 2100 bioanalyzer (Agilent Technologies, Palo Alto, CA, USA), and the RNA integrity was checked using RNase-free agarose gel electrophoresis. The mRNA was enriched using oligo(dT) beads (Epicentre, Madison, WA, USA). The complete mRNA was fragmented into short fragments, and reverse-transcribed into cDNA using NEBNext Ultra RNA Library Prep Kit for Illumina (NEB #7530, New England Biolabs, Ipswich, MA, USA). The purified double-stranded cDNA fragments were end repaired and ligated to Illumina sequencing adapters. Ligated fragments were subjected to size selection using agarose gel electrophoresis and polymerase chain reaction (PCR) amplified. The cDNA library was sequenced using Illumina Nova Seq 6000 by Gene Denovo Biotechnology Company, Guangzhou, China.

### 2.3. De Novo Assembly and Data Processing

Raw reads containing adapters, more than 10% of unknown nucleotides or low quality bases were removed, which affected the following assembly and analysis [18]. Additionally, the raw sequence reads were cleaned using the SolexaQA package. The short reads alignment tool, Bowtie2, was used for mapping reads to unigenes. The remaining clean reads were further used in assembly and gene abundance calculation [19]. The clean reads were further assembled and aligned with the sweetpotato genome data (http://public-genomesngs.molgen.mpg.de/cgibin/hgGateway?hgsid=9052&clade=plant&org=Ipomoea+batatas&db=ipoBat4, accessed on 9 August 2021).

### 2.4. Differential Expression Analysis (DEGs)

The fragments per kilobase of transcript per million fragments mapped (FPKM) value was calculated to quantify its expression abundance and variations for each gene using RSEM software. The reads data obtained through the analysis of gene expression levels were used as the input data for DEGs [20]. The differential expression values between XZ8 and X192 were estimated using DESeq2 software. The thresholds of |log2 fold change (FC)| ≥ 2 and the false discovery rate (FDR) < 0.01 were defined as screening criteria for DEG detection [21].

### 2.5. Gene Ontology (GO) and Kyoto Encyclopedia of Genes and Genomes (KEGG) Enrichment Analysis

Gene ontology (GO) and the Kyoto Encyclopedia of Genes and Genomes (KEGG) functional enrichment analysis provide a significant abundance of DEGs for biological function pathways [22]. All DEGs were integrated with the GO (http://www.geneontology.org/, accessed on 9 August 2021) and KEGG (http://www.genome.jp/kegg, accessed on 9 August 2021) databases [21]. The clusterProfiler R package and KOBAS software were implemented to analyze the statistical enrichment of the DEGs in GO and KEGG [23]. The calculated *p*-value was considered to be significantly different when *p* < 0.05.

### 2.6. Quantitative Real-Time PCR (qRT-PCR) Validation

Total RNA was extracted from two sweetpotato lines SR at 60, 90, 120, and 150 DAP using the RNApure Plant Kit (DNase I) (CWBIO, Beijing, China). cDNA was reverse-transcribed using the SuperScript II Kit (TaKaRa, Beijing, China) according to the manufacturer’s instructions. Four selected DEGs from the RNA-Seq were validated using quantitative real-time PCR (qRT-PCR) with the one-step real-time PCR System (Applied Biosystems, Foster, USA). The qRT-PCR of each reaction (total volume 20 μL) contained 10 μL of SYBR Master Mix (2×, (TaKaRa, Dalian, China), 1.0 μL of primers, 1.0 μL of the cDNA template, and 7 μL of RNase-free water. The 2^−ΔΔCT^ method was used to calculate the relative expression levels of genes [24]. The sweetpotato *tublin* gene was used as a reference. The PCR procedure was: 95 °C for 60 s, 40 cycles of denaturation at 95 °C for 15 s, annealing at 60 °C for 15 s, and elongation at 72 °C for 20 s, then a melting curve was generated and analyzed. All the primers used for the qRT-PCR validation are listed in Table S3. Three biological replicates were used in statistical analysis and the values in figures were means ± SD (standard deviation). Statistically significant differences at *p* < 0.05 and *p* < 0.01 were indicated by asterisks * and **, respectively.

## 3. Results

### 3.1. Sweetpotato SR Characteristics at Different Stages

**Figure 1:**
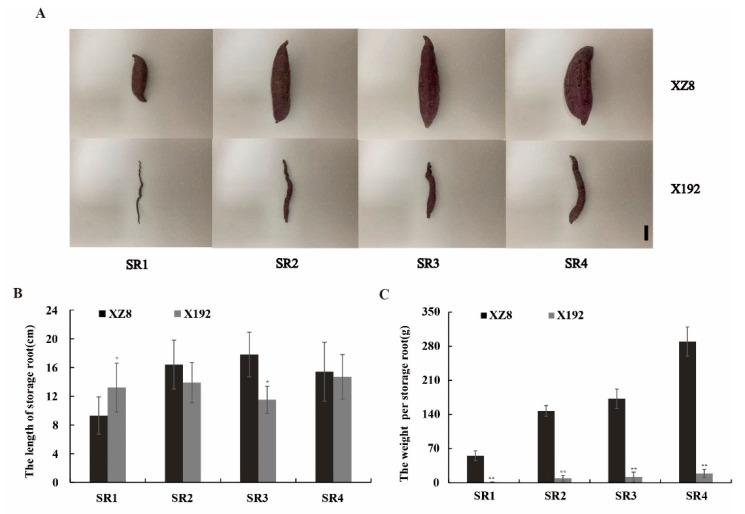
Phenotypic comparison of XZ8 and X192 at different SR expansion stages. (**A**) Phenotypic of XZ8 and X192 at different SR expansion stages. Scale bar, 4 cm. (**B**) The storage root length of XZ8 and X192 at different SR expansion stages. (**C**) The storage root weight of XZ8 and X192 at different SR expansion stages. Experiments were conducted in three biological replicates. Values are means ± SD (n = 3). Student’s *t*-tests, * *p* < 0.05, ** *p* < 0.01.

To understand the differences in the SR expansion of XZ8 and X192, two sweetpotato lines’ SRs at four distinct expansion stages (Considering the actual production value and the difference in transcriptome sequencing, we choose the four periods: 60, 90, 120, and 150 DAP) were harvested (Figure 1A). The experiment materials were randomly selected from 10 seedings in each row (we removed the first and the last one) and three replicates. The SRs of XZ8 expanded at SR1 whereas X192 maintained fibrous roots. Then, the SRs of XZ8 expanded significantly during the whole growth stage (Figure 1B,C). Additionally, at SR4, the fresh yield per SR of XZ8 was at a maximum of 289.64 g (Figure 1C). The SR of X192 began thickening at SR2, only 9.26g compared to 147.45 g for XZ8. At SR4, X192 gradually hardened and developed into SRs but each SR weighed less than 20 g (Figure 1C). These results indicated that there are extremely significant differences in the SRs development between the two sweetpotato lines and this still needs further research.

### 3.2. RNA-Seq Analysis

To compare and understand the molecular mechanism of SR at different expansion stages between XZ8 and X192, RNA-Seq analysis was performed at four expansion stages (60, 90, 120, and 150 DAP) in three biological replicates. A total of 24 cDNA libraries were constructed (2 lines×4 sampling time points×3 biological replicates, i.e., XZ8-1 and X192-1, SRs harvested at 60 DAP of XZ8 and X192; XZ8-2 and X192-2, SRs harvested at 90 DAP of XZ8 and X192; XZ8-3 and X192-3, SRs harvested at 120 DAP of XZ8 and X192; XZ8-4 and X192-4, SRs harvested at 150 DAP of XZ8 and X192). After sequencing using Illumina Nova Seq 6000, more than 1200,000,000 raw data were obtained, of which approximately 99% were clean reads (Table S1). 

The clean reads were then aligned to the sweetpotato genome (http://public-genomes-ngs.molgen.mpg.de/cgi-bin/hgGateway?hgsid=9052&clade=plant&org=Ipomoea+batatas&db=ipoBat4, accessed on 9 August 2021) using HISAT [25]. A total of 57,043 unigenes were produced and more than 80% annotated, except for X192-4 (Table S2). Genes that were not included in the reference genome (or collection of reference genes) are defined as novel genes, and 7940 of these genes were detected (Table S2).

**Figure 2:**
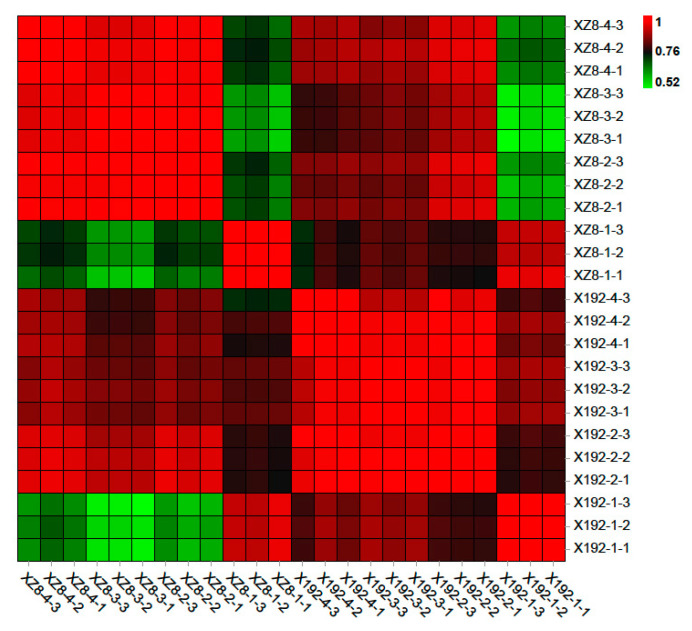
Correlation heatmap analysis of 24 samples. The x-axis and y-axis are for each sample, and the color depth represents the correlation coefficient of the samples. The deeper the red color, the more correlated the samples are. The deeper the green color, the less correlated the samples are.

Based on the results of the expression quantity of each sample, principal component analysis (PCA) and heatmap were used to reveal the repeatability between samples and the correctness of sequencing. Using R (http://www.r-project.org/, accessed on 9 August 2021) to carry out PCA, the distance relationship between samples was studied with the purpose of dimensionality reduction [26]. This method can effectively find the most major elements and structures in the data by means of variance decomposition, and reflect the complex sample composition relationship to the two characteristic values of the horizontal and vertical coordinates, so as to achieve the effect of finding the 24 samples’ aggregation rule from the sequencing data (Figure S1). As shown in Figure S1, the biological replicates from the same treatment group were highly correlated, only X192-4 showed as slightly less correlated. The results of the heatmap coincided with our PCA and visually showed the correlation between 24 samples in the form of a heatmap (Figure 2). In addition, the results showed that XZ8-2, XZ8-3 and XZ8-4 were highly correlated, which means that the gene expression profiles in the three stages were closer (Figure S1 and Figure 2). 

### 3.3. Identification of DEGs and Cluster Analysis

**Figure 3:**
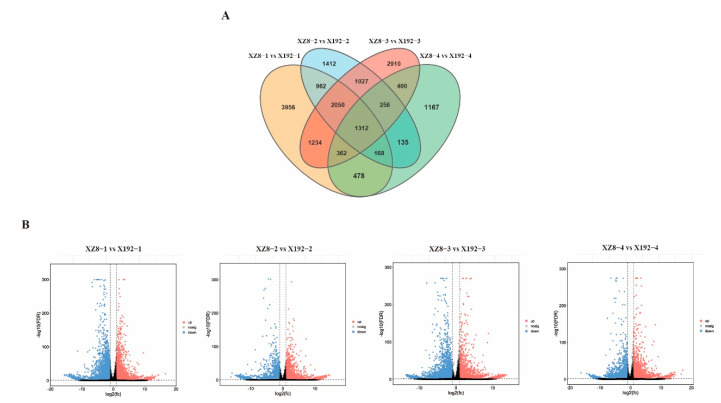
Total number of DEGs in XZ8 and X192 in the four groups. (**A**) Venn diagram of DEGs in XZ8 and X192 in the four groups (XZ8-1 vs. X192-1, XZ8-2 vs. X192-2, XZ8-3 vs. X192-3 and XZ8-4 vs. X192-4). The numbers in each circle represent the number of DEGs in the corresponding group. The overlapped part of the circle represents the common DEGs between the groups. (**B**) Volcano plots of XZ8 and X192 in the four groups. The x-axis represents log base two-fold change, the y-axis represents –logbase 10 Q-value (*p*-adjusted) for each plot. The DEGs were indicated by the red dots (up-regulated) and the blue dots (down-regulated), and genes without significant difference were indicated by the gray dots.

Differential expression analysis was performed using DESeq2 with the parameter of FDR below 0.05 and absolute FC ≥ 2. The number of DEGs was identified in the four groups (XZ8-1 vs. X192-1, XZ8-2 vs. X192-2, XZ8-3 vs. X192-3 and XZ8-4 vs. X192-4) and these were 10,522, 7286, 9551 and 4278. Of these, only 1312 DEGs were specifically expressed in the four groups (Figure 3A). At the stage of XZ8-4 vs. X192-4, the number of DEGs was significantly decreased on account of the SR expansion period being finished (Figure 3A). Moreover, the distribution of DEGs in the four groups was illustrated as volcano plots. The up-regulated DEGs are indicated by red dots; the down-regulated DEGs are indicated by orange dots and non-DEGs are indicated by blue dots (Figure 3B).

### 3.4. Gene Enrichment Analysis

**Figure 4:**
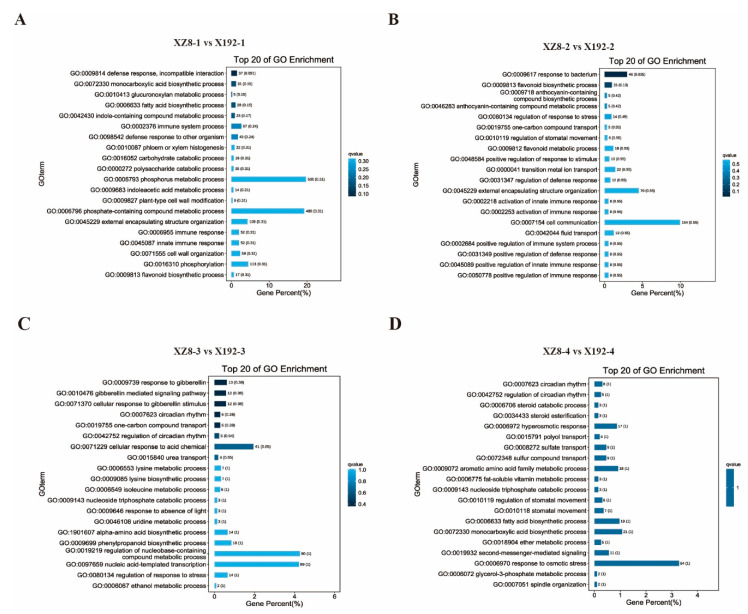
Functional classification of DEGs according to GO enrichment analysis in XZ8 and X192 in the four groups. (**A**–**D**) XZ8-1 vs. X192-1, XZ8-2 vs. X192-2, XZ8-3 vs. X192-3 and XZ8-4 vs. X192-4. x-axis: the percentage of genes enriched in this process in total annotated genes; y-axis: name of the GO enrichment terms. The color depth represents the Q value. The darker the color, the smaller the Q value and the higher the enrichment degree.

To provide further insight into which pathways the DEGs involved, DEGs were mapped to each item of the GO database (http://www.geneontology.org/, accessed on 9 August 2021). Ggplot2 software was used to calculate the number of DEGs and categorize DEGs in the GO enrichment analysis [27]. Based on the GO categories, obtained using gene list differences in GO function statistics, the top 20 GO enrichment terms of the four groups (XZ8-1 vs. X192-1, XZ8-2 vs. X192-2, XZ8-3 vs. X192-3 and XZ8-4 vs. X192-4) were listed (Figure 4). It should be noted that DEGs in the four groups were significantly enriched in different processes. The DEGs in XZ8-1 vs. X192-1 were significantly enriched in two processes: ‘phosphorus metabolic’ and ‘phosphate-containing compound metabolic’ (Figure 4A). A total of 154 DEGs in XZ8-2 vs. X192-2 were classified as ‘cell communication’ (Figure 4B). The DEGs in XZ8-3 vs. X192-3 and XZ8-4 vs. X192-4 were found to be mainly involved in the ‘regulation of nucleobase-containing compound metabolic process’, ‘nucleic acid-templated transcription’ and ‘response to osmotic stress’, respectively (Figure 4C,D). 

**Figure 5:**
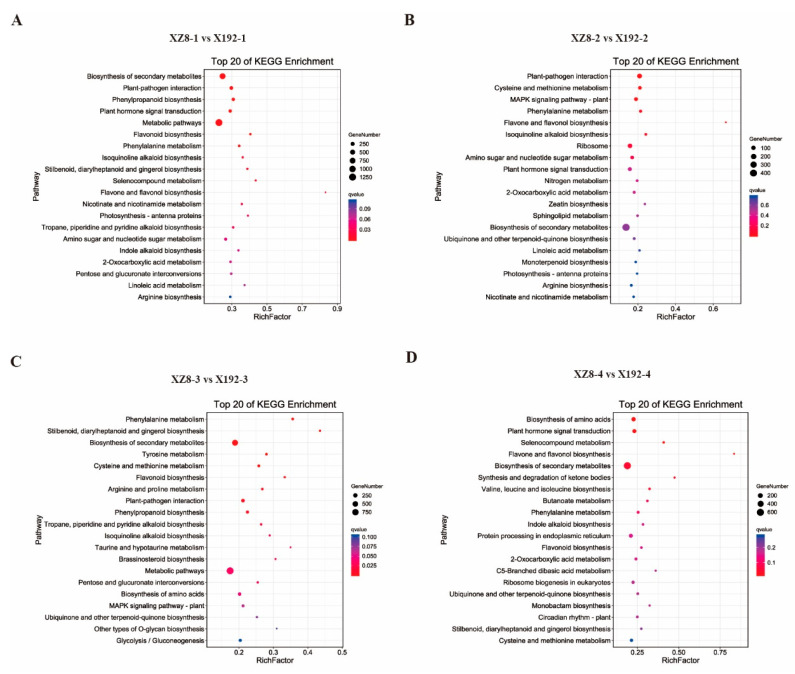
The top 20 of KEGG enrichment pathways in XZ8 and X192 in the four groups. (**A**–**D**) XZ8-1 vs. X192-1, XZ8-2 vs. X192-2, XZ8-3 vs. X192-3 and XZ8-4 vs. X192-4. The y-axis is the pathway, and the x-axis is the percentage of this pathway of the total Rich Factor. The color depth represents the Q value. The darker the color, the smaller the Q value and the higher the enrichment degree. The size of the dots indicates the number of DEGs in this pathway.

In addition, we conducted a KEGG analysis using the top 20 pathways with the lowest Q values (Figure 5). KEGG analysis revealed that the most predominant subcategory among various pathways was ‘biosynthesis of secondary metabolites’ and ‘metabolic pathways’ with enrichment in the four groups, followed by ‘plant-pathogen interaction’, ‘biosynthesis of amino acids’ and ‘plant hormone signal transduction’ (Figure 5A–D).

### 3.5. Validation of DEGs during SR Expansion

**Figure 6:**
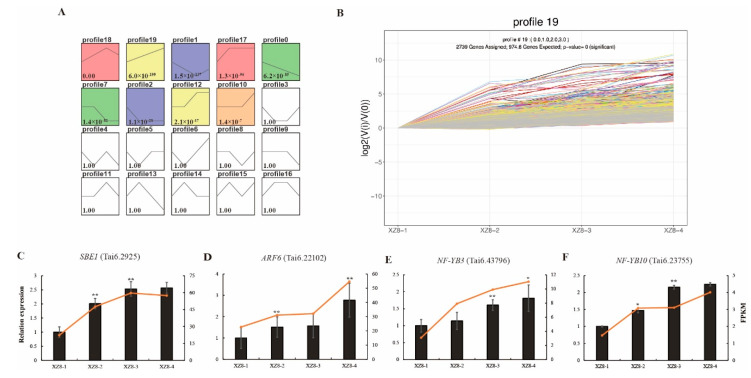
Trend analysis of genes across the different SR expansion stages of XZ8. (**A**) Trend analysis of genes at four SR expansion stages. The black line represents the trend line. The number represents the *p* value. (**B**) Significantly enriched trend analysis of profile 19. The lines represent genes. The y-axis is log2 value, and the x-axis is the four stages of XZ8. (**C**) The trend of *SBEI* (starch branching enzyme I, Tai6. 2925) expression in qRT-PCR and transcriptome. (**D**) The trend of *ARF6* (auxin response factor 6, Tai6. 22102) expression in qRT-PCR and transcriptome. (**E**) The trend of nuclear factor-Y transcription factor *NF-YB3* (Tai6. 43796) expression in qRT-PCR and transcriptome. (**F**) The trend of nuclear factor-Y transcription factor *NF-YB10* (Tai6. 23735) expression in qRT-PCR and transcriptome. Black bars represent the data of qRT-PCR and red lines represent the data of transcriptome. FPKM values were used to represent the relative expression of genes in the transcriptome. Values are means ± SD (n = 3). Student’s *t*-tests, * *p* < 0.05, ** *p* < 0.01.

Trend analysis is a method widely used in transcriptomes for clustering gene expression patterns and identifying candidate genes. The curve in multiple stages is based on the characteristics of multiple consecutive samples (at least three) containing specific time, space or treatment between the samples [28]. Thus, combined with a relationship analysis of the samples, we used the enrichment trend method to analyze the variation in different genes at the four expansion stages of XZ8. A total of 31,637 DEGs were studied and divided into 20 profiles according to the expression level pattern (Figure 6A). It is remarkable that profiles sorted by *p* value in ascending order and profiles that have colors represent significant enrichment, which should receive the highest priority. The same color represents the same trend, such as profile 0 and profile 7, profile 1 and profile 2, profile 12 and profile 19, profile 17 and profile 18. Profiles without color represent insignificant enrichment of the trend or too few genes enrichment (Figure 6A). GO enrichment analysis of the four stages more enriched in ‘external encapsulating structure organization’, ‘cell wall organization or biogenesis’, ‘tissue development’, ‘response to endogenous stimulus’ and ‘response to acid chemical’ (Figure S2A). KEGG pathways analysis of the four stages showed that 31,637 genes were mostly mapped in ‘biosynthesis of secondary metabolites’ and ‘metabolic pathways’, this result is the same as the KEGG enrichment in each group (Figure S2B). 

In profile 19, the expression level of all genes continued to increase with the time of SR expansion, indicating that these genes have always maintained high expression during SR expansion (Figure 6B) and thus aid in the adaptation to SR expansion. Therefore, we focused on the gene expression in profile 19. First, GO categories analysis showed that 2196 genes in profile 19 were significantly enriched in the regulation of the gene expression process (Figure S3A). Meanwhile, our KEGG pathways analysis revealed genes in profile 19 were majority mapped in five pathways, including ‘valine, leucine and isoleucine degradation’, ‘peroxisome’, ‘glycerophospholipid metabolism’, ‘ubiquitin mediated proteolysis’ and ‘plant hormone signal transduction’ (Figure S3B).

To validate the results of RNA-seq and trend analysis, we conducted a qRT-PCR analysis on the transcript levels of four DEGs, including the starch biosynthesis-related gene *SBEI* (starch branching enzyme I, Tai6. 2925), transcription factors of *ARF6* (auxin response factor 6, Tai6. 22102), nuclear factor-Y transcription factor *NF-YB3* (Tai6. 43796) and *NF-YB1*0 (Tai6. 23735) as potential candidates regulating SR expansion. We set the value to 1 of XZ8-1; the results showed that expression levels of *SBEI*, *ARF6*, *NF-YB3* and *NF-YB10* were all up-regulated during SR expansion (Figure 6C–F). Overall, the transcript expression profiles obtained from qPCR were completely consistent with those obtained in the transcriptome analysis and further confirmed the reliability of our RNA-Seq.

## 4. Discussion

SR is the main nutritional and edible organ, and directly determines the quality and quantity of sweetpotato [29]. However, SR expansion is a complex physiological process that is affected by many factors, including internal and external environments. In this study, our team selected a new sweetpotato line from the segregation population of Xuzishu 8 (XZ8) and Meiguohong, called Xu 18-192, X192. Its SR expansion was delayed significantly, but its other traits were not different from XZ8 (Figure 1A). Before the harvest of 60 DAP, the SR of XZ8 had expanded, but X192 had not developed. Moreover, the SR of XZ8 were larger and heavier than X192 during the whole growth stage (Figure 1). To elucidate the mechanism of SR expansion in XZ8 and X192, we performed a comparative transcriptome analysis. The changes in gene expression during the four stages (XZ8-1 vs. X192-1, XZ8-2 vs. X192-2, XZ8-3 vs. X192-3 and XZ8-4 vs. X192-4) were studied. A total of 57,043 genes were identified, and the obtained genes were annotated to the NR, KOG, Pfam, Swiss-Prot and GO databases (Tables S1 and S2; Figure 3). GO analysis revealed that the DEGs in the four groups were enriched in various processes, including ‘phosphorus metabolic’, ‘phosphate-containing compound metabolic’, ‘cell communication’ and ‘reproduction’ (Figure 4). KEGG analysis revealed that these DEGs were mainly involved in two pathways, ‘biosynthesis of secondary metabolites’ and ‘metabolic’ (Figure 5).

Starch is the main component of SR and accounts for approximately 50%~80% of the dry weight of sweetpotato SR; its metabolic activity significantly affected SR expansion. Starch accumulated continuously at the initial SR expansion stage and increased gradually during the whole stage of SR expansion, and only slightly decreased at the later stage of SR expansion [30]. With the increase in endogenous sucrose, the activity of the *IbAGP1* (ADP-glucose pyrophosphorylase subtypes) promoter increased, while the activity of the *IbAGP2* promoter decreased significantly. *IbAGP2* was mainly expressed in the early stage of SR expansion and *IbAGP1* was largely expressed in the late stage of SR expansion, suggesting that both of them play a certain role in the SR expansion of sweetpotato [31]. The *BMY11* (*β-amylase 11*) gene orthologous to *Arabidopsis β-amylase 1*, lysed starch granules and recovered to synthesize larger granules, which promoted starch accumulation and SR expansion [32]. The *IbSnRK1* (sucrose non-ferment-1-related protein kinase-1) gene can improve the expression level of genes related to the starch biosynthesis pathway and the activities of key enzymes, and affect the starch content of transgenic sweetpotato. Additionally, it is beneficial to improve the quality and yield of sweetpotato [33]. Therefore, we selected *SBEI* (starch branching enzyme I), a DEG involved in starch metabolism, from profile 19 of the trend analysis (Figure 6A,B). *SBEI* is a key enzyme in starch synthesis that regulates the proportion of amylose and amylopectin, and directly affects the structure and physical properties of starch granules [34]. The quantitative RT-PCR results indicated that *SBEI* was strongly up-regulated during SR expansion, especially in XZ8-2 (Figure 6C). These results indicate that *SBEI* might play a critical role in SR expansion by regulating the formation and content of starch.

Transcription factors can specifically bind to cis-acting elements in the promoter region of eukaryotic genes to induce or inhibit the transcription of the downstream targets [35]. The development of SR or tuber in tuber crops mostly relies on transcription factor regulation, including starch metabolism and hormone biosynthesis and transport [17]. In recent years, some transcription factors related to the SR expansion of sweetpotato have been identified. MADS-box and homeobox transcription factors have been studied extensively in tuber crops [36]. MADS-box proteins are highly conserved transcription factors widely distributed in eukaryotes, involved in the regulation of flower organ formation, flowering time, fruit ripening, root growth and development [37,38]. In potato, MADS-box gene *StMADS11* was highly expressed in developing tubers, suggesting that this gene may be involved in regulating tuber development [39]. The decreased expression of *POTM1* caused lateral bud growth and a drop in tuber yield. Further studies conclusively showed that *POTM1* regulates the growth of lateral buds and tuber yield by up-regulating the level of cytokinin in transgenic plants [40]. The evolutionary and functional differentiation of MADS-box family members in potato revealed that *StMADS1* and *StMADS13* are likely involved in tuber development as downstream targets of *StSP6A* [41]. *IbMADS3* and *IbMADS4* genes were highly expressed in the vegetative organs and at the beginning of the SR expansion of sweetpotato, suggesting that they might be positive regulators in SR expansion [42]. In potato, overexpression of the *IbMADS1* gene activated root xylem differentiation and thickened adventitious roots. The results indicate that *IbMADS1* has a function in controlling SR expansion [43].

Homeobox is a homologous heteromorphic transcription factor that plays an important role in plant growth and development [44]. The overexpression of *POTH1* (a member of the homeobox family) in potatoes can promote tuber formation. Further studies showed that the expression of *GA1* and *GA20* genes decreased and the expression of *GA19* increased in transgenic plants, indicating that the *POTH1* gene affected tuber development by regulating the level of GA [45]. The overexpression of *STBEL5* accelerated the formation of tuber. The GA level decreased continuously along with the enhanced level of CTK. Both *POTH1* and *StBEL5* can directly interact with the promoter region of *GA20ox1*, regulating the synthesis of GA and affecting tuber development [46]. *Ibkn1*, *Ibkn2* and *Ibkn3* genes were cloned from sweetpotato and were all highly expressed during SR expansion. Further analysis suggested that they might affect SR expansion by regulating the level of CTK [47].

Using the above results, we identified three differentially expressed transcription factors from trend analysis (Figure 6D–F). *ARF6* (Tai6. 22102) was an auxin response factor that specifically binds to AuxRE in the gene promoter to activate or inhibit the expression level [48]. The expression levels of the *ARF6* gene showed a gradual upregulation during SR expansion, and expression was significantly increased in XZ8-4 (Figure 6D). In addition, our qPCR results revealed that the expression of two nuclear factor-Y transcription factors, *NF-YB3* (Tai6. 43796) and *NF-YB10* (Tai6. 23735) [49], were also increased in SR expansion stages (Figure 6E,F). These results indicate that *ARF6, NF-YB3 and NF-YB10* might act as enhancers in SR expansion.

## 5. Conclusions

The key to increasing the yield of sweetpotato lies in the formation and expansion of SR. In this study, we characterized a comparative transcriptome of different SR stages of Xuzishu 8 and Xu 18-192 using Illumina sequencing technology. The results of de novo assembly identified and annotated 57,043 unigenes, of which there were 31,637 DEGs. A combination of GO and KEGG analyses, as well as trend analysis, showed starch metabolism and transcription factors have a significant and continuous effect on the SR expansion of sweetpotato. The four key genes *SBEI*, *ARF6*, *NF-YB3* and *NF-YB10* were proved as potential candidates for regulating SR expansion. The identification of these genes remains a challenging goal in gene engineering research and further research is needed to certify their functions. Thus, our present findings improve our understanding of the molecular mechanism underlying SR expansion and could facilitate the breeding of high-yield lines of sweetpotato.

## Data Availability

RNA-seq data will soon be submitted to the NCBI Sequence Read Archive (SRA). Additional data supporting the findings are included in the article.

## Supplementary Materials

The following supporting information can be downloaded at: https://www.mdpi.com/article/10.3390/genes13071156/s1, Supplementary Table S1. The reads of de novo transcriptome assembly of sweetpotato lines. Supplementary Table S2. Numbers of unigenes annotated using reference genome. Supplementary Table S3. Primers used in real-time PCR. Supplementary Figure S1. Principal component analysis (PCA) of 24 samples. Supplementary Figure S2. Enriched distributions of DEGs in GO categories according to GO enrichment analysis in XZ8 at the four SR expansion stages and profile 19. Supplementary Figure S3. The top 20 of KEGG enrichment pathways in XZ8 at the four SR expansion stages and profile 19.

## Abbreviations

ABA: abscisic acid; ARF, auxin response factor; BMY11, β-amylase 11; CTK, cytokinin; DAP, days after planting; DEG, differentially expressed gene; DNB, dynamic network biomarker; FC, fold change; FDR, false discovery rate; GA, gibberellin; GO, gene ontology; IAA, auxin indole-3-acetic acid; JA, jasmonic acid; KEGG, Kyoto Encyclopedia of Genes and Genomes; KOG, euKaryotic Orthologous Groups; NCBI, National Center for Biotechnology Information; PCA, principal component analysis; PCR: polymerase chain reaction; qRT-PCR, quantitative real-time PCR; RNA-Seq, RNA sequencing; FPKM, fragment per kilobase of transcript per million; SBEI, starch branching enzyme I; SD, standard deviation; SR, storage root; SRF1, storage root formation 1; TAIR, The Arabidopsis Information Resource.
