# Supplementary material for: Comparative Transcriptome Profiling Reveals the Genes Involved in Storage Root Expansion in Sweetpotato (Ipomoea batatas (L.) Lam.)
Source: Genes (Basel). 2022 Jun 27;13(7):1156. doi: 10.3390/genes13071156 (PMC9321896; doi:10.3390/genes13071156)

Fig S1

Principal component analysis (PCA) at 24 samples. The PC1 coordinate represents the first principal component, and the percentage in brackets represents the contribution value of the first principal component to the sample difference. The PC2 coordinate represents the second principal component, and the percentage in brackets represents the contribution value of the second principal component to the sample difference. The colored dots in the figure represent each sample.

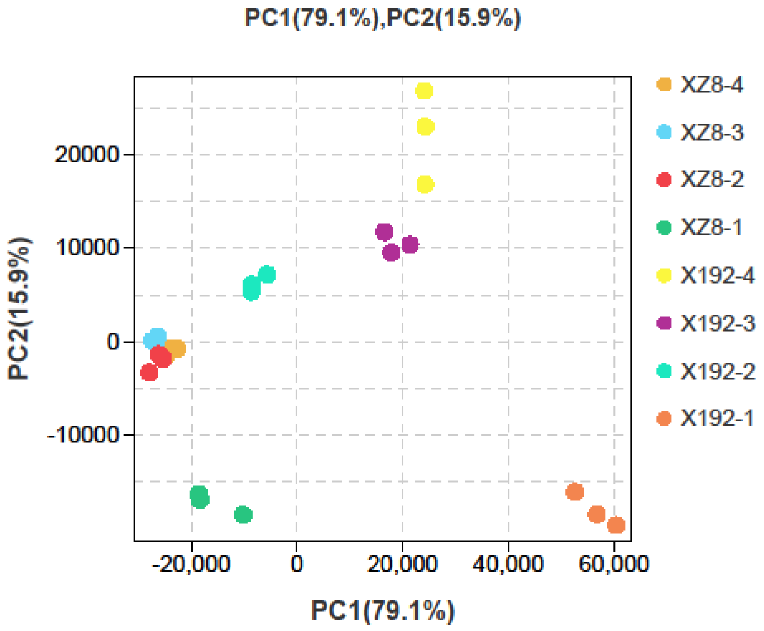

Supplement: Supplementary file 1 [file genes-13-01156-s001.zip › Supplementary Figure S1.pdf]
