# Supplementary material for: Comparative Transcriptome Profiling Reveals the Genes Involved in Storage Root Expansion in Sweetpotato (Ipomoea batatas (L.) Lam.)
Source: Genes (Basel). 2022 Jun 27;13(7):1156. doi: 10.3390/genes13071156 (PMC9321896; doi:10.3390/genes13071156)

Fig S2

Enriched distributions of DEGs in GO categories according to GO enrichment analysis in XZ8 at the four SR expansion stages and profile 19. (A) Enriched distributions of DEGs in GO categories in XZ8 at the four SR expansion stages. (B) Enriched distributions of DEGs in GO categories according to GO enrichment analysis in profile 19. x-axis: The percentage of genes enriched to this process ; y-axis: Name of the GO term. The color depth represents the Q value. The darker the color, the smaller the Q value and the higher the enrichment degree. The black circles are the number of genes in this pathway. The size of the black circle represents the number of genes.

A

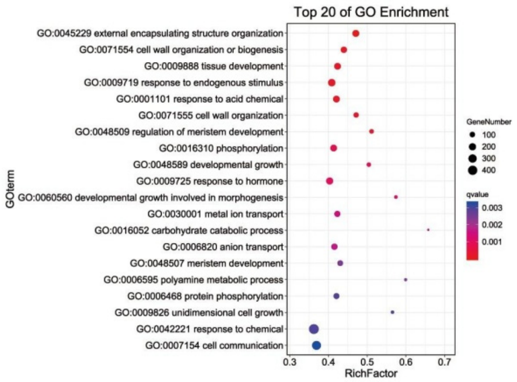

B

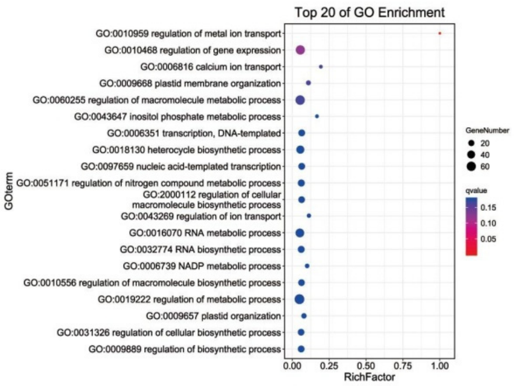

Supplement: Supplementary file 1 [file genes-13-01156-s001.zip › Supplementary Figure S2.pdf]
