# Supplementary material for: Comparative Transcriptome Profiling Reveals the Genes Involved in Storage Root Expansion in Sweetpotato (Ipomoea batatas (L.) Lam.)
Source: Genes (Basel). 2022 Jun 27;13(7):1156. doi: 10.3390/genes13071156 (PMC9321896; doi:10.3390/genes13071156)

Fig S3

The top 20 of KEGG enrichment pathways in XZ8 at the four SR expansion stages and profile 19. (A) The top 20 of KEGG enrichment pathways in XZ8 at the four SR expansion stages. (B) The top 20 of KEGG enrichment pathways in profile 19. The y-axis is pathway, and the x-axis is the percentage of this pathway. The color depth represents the Q value. The darker the color, the smaller the Q value and the higher the enrichment degree. The black circles are the number of genes in this pathway. The size of the black circle represents the number of genes.

A B

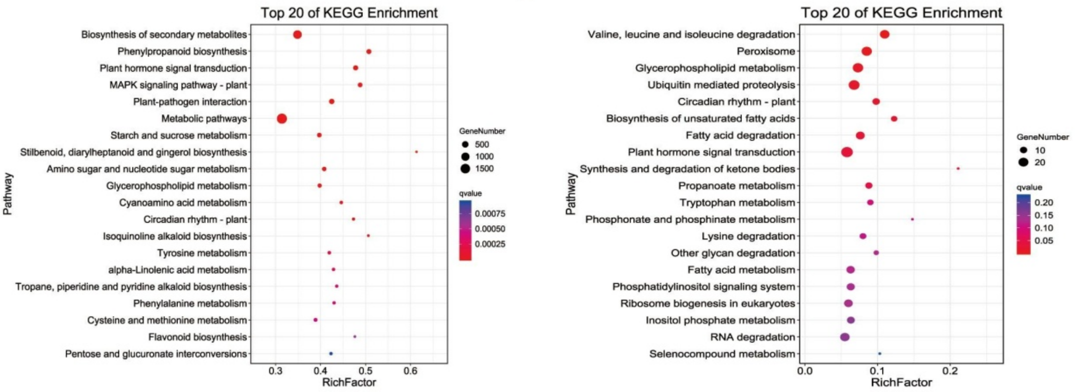

Supplement: Supplementary file 1 [file genes-13-01156-s001.zip › Supplementary Figure S3.pdf]
