# Supplementary material for: Comparative Transcriptome Profiling Reveals the Genes Involved in Storage Root Expansion in Sweetpotato (Ipomoea batatas (L.) Lam.)
Source: Genes (Basel). 2022 Jun 27;13(7):1156. doi: 10.3390/genes13071156 (PMC9321896; doi:10.3390/genes13071156)
Supplement: Supplementary file 1 [file genes-13-01156-s001.zip › Supplementary Table S1.pdf]

Table S1

The reads of de novo transcriptome assembly of sweetpotato lines.

Sample	RawDatas	CleanData	Q20 (%)	Q30 (%)	Adapter	LowQuality
XZ8-1	154081074	153385768	97.83	93.85	43680	651626
XZ8-2	137278960	136671032	97.89	94.03	50130	557798
XZ8-3	154857374	143230448	97.78	93.74	46894	561776
XZ8-4	145473570	144858842	97.88	93.96	43518	571210
X192-1	169993496	169031730	97.78	93.82	59166	902600
X192-2	154857374	154153232	97.84	93.89	47974	656168
X192-3	167072040	166355406	97.91	94.03	44204	672430
X192-4	170065218	169181118	97.82	93.86	73812	810288

RawDatas: total number of reads. CleanData: number of high-quality reads. Q20 (%): The number of bases whose quality value reached above Q20 level and the percentage of them in RawData (or CleanData). Q30 (%): The number of bases whose quality value reached above Q30 level and the percentage of them in RawData (or CleanData).Adapter: number of reads that contain adapter. LowQuality: number of low quality reads.
