# Supplementary material for: Comparative Transcriptome Profiling Reveals the Genes Involved in Storage Root Expansion in Sweetpotato (Ipomoea batatas (L.) Lam.)
Source: Genes (Basel). 2022 Jun 27;13(7):1156. doi: 10.3390/genes13071156 (PMC9321896; doi:10.3390/genes13071156)
Supplement: Supplementary file 1 [file genes-13-01156-s001.zip › Supplementary Table S2.pdf]

Table S2

Numbers of unigenes annotated using reference genome.

Sample	Refer genes	Sequenced refer genes	sequenced_novel genes	Total_genes
XZ8-1	49063	41316	5834	47150
XZ8-2	49063	40605	5609	46214
XZ8-3	49063	40445	5493	45884
XZ8-4	49063	40287	5424	45711
X192-1	49063	40342	5502	45844
X192-2	49063	40134	5413	45547
X192-3	49063	39663	5269	44932
X192-4	49063	38049	4788	42837
