# Supplementary material for: Comparative Transcriptome Profiling Reveals the Genes Involved in Storage Root Expansion in Sweetpotato (Ipomoea batatas (L.) Lam.)
Source: Genes (Basel). 2022 Jun 27;13(7):1156. doi: 10.3390/genes13071156 (PMC9321896; doi:10.3390/genes13071156)
Supplement: Supplementary file 1 [file genes-13-01156-s001.zip › Supplementary Table S3.pdf]

Table S3

Primers used in real-time PCR.

Primer	Primer sequence	Purpose
SBEI-F	5'- GGTGGGCCATGATGTAGACCATTT -3'	Real-time PCR
SBEI-R	5'- CTGGCAGGCTGTTCATCTTGGACT -3'	Real-time PCR
ARF6-F	5'- TTCTTCTTGGCGATGACCCC -3'	Real-time PCR
ARF6-R	5'- ATCCAACAGATGCCATGCCA -3'	Real-time PCR
NF-YB3-F	5'- GGACAGGTTCCTGCCGATAG -3'	Real-time PCR
NF-YB3-R	5'- GCCGTTGATCGTCTTCCTCT -3'	Real-time PCR
NF-YB10-F	5'- TCTTGACCCCCTGAAGGTGT -3'	Real-time PCR
NF-YB10-R	5'- GCTGACCCTGTGAGTTTCCA -3'	Real-time PCR
tublin-F	5'- CAACTACCAGCCACCAACTGT -3'	Real-time PCR
tublin-R	5'- CAAGATCCTCACGAGCTTCAC -3'	Real-time PCR
